# Loss of Nuclear Functions of HOXA10 Is Associated With Testicular Cancer Proliferation

**DOI:** 10.3389/fonc.2018.00594

**Published:** 2018-12-07

**Authors:** Ruiqi Chen, Haolong Li, Yinan Li, Ladan Fazli, Martin Gleave, Lucia Nappi, Xuesen Dong

**Affiliations:** ^1^Department of Urologic Sciences, Vancouver Prostate Centre, The University of British Columbia, Vancouver, BC, Canada; ^2^Department of Medicine, King's College Circle Toronto, University of Toronto, Toronto, ON, Canada

**Keywords:** testicular cancer, testicular germ cell tumor, HOXA10, TP53, proliferation

## Abstract

**Background:** HOXA10 is a key transcriptional factor that regulates testis development as reported from previous transgenic mouse models and human inherited diseases. However, whether it also plays important roles in promoting the development of testicular cancer is not well-understood.

**Objective:** To study the expression of HOXA10 and its regulated signaling pathways in testicular cancers.

**Design, Setting, and Participants:** A tissue microarray was constructed with benign and cancerous testis. TCam2, NT-2, and NCCIT cell models were applied in this study.

**Intervention:** Immunohistochemistry and immunofluorescence were performed to measure the expression and cellular localization of HOXA10 in testicular cancer tissues and cell models. Cell proliferation and cell cycling rates were determined by BrdU incorporation and flow cytometry assays. HOXA10 transcriptomes were profiled with Ampliseq RNA-seq in testicular cancer cells. Immunoblotting assays were used to detect HOXA10-regulated signaling.

**Results:** HOXA10 is a nuclear protein in benign spermatocytes. Reduced nuclear expression and increased cytoplasmic expression of HOXA10 are associated with testicular cancers. These changes are consistent in both seminoma and non-seminoma. Enhanced HOXA10 expression in testicular cancer cell models inhibits cell proliferation and delays cell cycle progression through G2/M phases. These functions of HOXA10 mainly affect the TP53, cKit, STAT3, AKT, and ERK signaling pathways.

**Conclusions:** Loss of nuclear functions of HOXA10 enhances proliferation of testicular cancer cells, suggesting that downregulation of HOXA10 transcription activity may promote the development of testicular cancers.

## Introduction

Testicular germ cell tumor (TGCT) is the most common form of cancer in young men aged between 15 and 40 and the incidence has been increasing over the past few decades worldwide (1–3). Despite a relatively high cure rate of 95% in all TGCT patients upon diagnosis and 80% in patients with metastatic TGCT, over 10,000 deaths worldwide were estimated in men with this disease (WHO GLOBOCAN 2012). Unlike spermatocytes, TGCT likely develops from gonocytes that failed to differentiate into spermatogonia, thus showing similar patterns of gene expression to those of stem cells and intratubular germ-cell neoplasm (4). TGCT can be categorized into seminoma, non-seminoma and spermatocytic tumors (5). Seminomas resemble closely with intratubular germ-cell neoplasia in a blocked differentiation state. Non-seminomas include several histological subtypes such as embryonal carcinoma, choriocarcinomas, yolk-sac tumors, and teratoma. Of note, choriocarcinomas and yolk-sac tumors present with extraembryonal differentiations while teratomas show somatic differentiations that contain all three germ layers.

Although previous studies have identified some genetic and epigenetic aberrations associated with TGCT, one group of genes that may play important roles in this process is the homeobox gene family. The homeobox gene family consists of 37 genes that encode transcription factors to regulate morphogenesis and differentiation of cells during embryogenesis, and are frequently deregulated in various types of cancer (6). These genes are crucial in the development of testis and expressed in human testis throughout adulthood (7). Importantly, TGCT tumorigenesis resembles early embryogenesis in many aspects ranging from gene expression patterns (8, 9), imprinting patterns (10, 11), and response to *in vitro* differentiation stimulus (12). Moreover, a previous epigenetic study showed that the promoter of some homeobox genes such as *RASSF1A, SCGB3A1*, and *HOXA9* were hypermethylated in testicular cancer tumors (13), further supporting that deregulation of homeobox proteins may contribute to the development of TGCT. Among the homeobox family genes, aberrant *HOXA10* expressions have been implicated in numerous other types of cancers but not yet described in TGCT. *HOXA10* is a member of the *abdominal B* class that also consists of *HOXA9* and *HOXA13* (14). Like other HOX family proteins, the HOXA10 protein is known to be localized in the nucleus and binds to DNA via a consensus core of TTAT/TTAC that is influenced by flanking sequences (15), interacting proteins such as MEIS and PBX (16, 17), and coregulatory proteins like histone deacetylase 2 (18). In the setting of cancers, *HOXA10* deregulation is known to play significant roles in mammary carcinoma, endometrial carcinoma, head and neck squamous cell carcinoma (HNSCC). Interestingly, the roles of *HOXA10* are complex among different types of cancers. For example, *HOXA10* overexpression promotes endometrial cancer and HNSCC activities (19, 20), whereas *HOXA10* inhibition is associated with breast cancer tumorigenesis (21). However, the role of *HOXA10* in TGCT has not yet been elucidated. In this study, we have combined tumor histological explorations, transcriptomic studies in cell lines, and functional investigations to characterize HOXA10 expression and function in TGCT tumorigenesis.

## Materials and Methods

### Human Testicular Samples

Human testicular tissue samples were obtained from the Vancouver Prostate Center (VPC) tissue bank at the University of British Columbia. Patient information is listed in Table S1. All patients have signed an informed consent to a protocol that was reviewed and approved by the UBC Clinical Research Ethics Board (Certificate #: H09-01628).

### Immunohistochemistry

Whole sections of testicular samples were fixed in 10% neutral buffered formalin, embedded in paraffin, stained with H&E, and evaluated by a pathologist (L.F.) for benign and cancerous portions of the testes. A tissue microarray (TMA) was also constructed, as previously described (22–24). Immunohistochemistry assays were performed by Ventana Discovery XT autostainer (Ventana). Slides in citrate buffer (pH = 6) were heated in a steamer for 30 min. After cooling for 30 min and washing, the slides were incubated in 3% H2O2 for 10 min, blocked with 3% BSA for 30 min, and then incubated with indicated primary antibodies for 2 h at room temperature. The slides were washed extensively with PBS and examined with UltraMap kit (Ventana). The sections were counterstained with hematoxylin and mounted with coverslips using the xylene-based mounting medium, Cytoseal (Stephen Scientific, Riverdale, NJ). Normal IgG antibodies (Santa Cruz) were used as negative controls. Information on HOXA10 and AR antibodies used in this study is listed in the Supplementary Materials. Stained slides were scanned by a Leica SCN400. Digital images were evaluated and scored by the pathologist (L.F), based on subcellular localization, intensity and percentile of positive cells within a tissue core. Digital images were evaluated by Dr. Ladan Fazli as well as by using the software, Image Pro Plus (Media Cybernetics Inc), to score the percentage of stained cells (0–16, 17–33, 34–66, and 67–100%, as 0–3 scores) and the staining intensity (no staining, low, moderate, and high intensity staining, as 0–3 scores). The histology index of HSCORE = ∑pi(*i* + 1), where *i* = the intensity of staining and pi = the percentage of stained cells as reported (22–24).

### Immunofluorescence Microscopy

Immunofluorescence assays were performed as previously described (25). Slides were deparaffinized, rehydrated through a series of graded alcohols, and washed in double deionized water for 5 min. Tissues were then placed in antigen unmasking solution (Vector Labs, Burlingame, CA), and antigen retrieval was performed by microwaving samples. Slides were cooled to room temperature, and washed with PBS (pH 7.4). For sequential immunofluorescence staining, the AR (N-20) antibody (#sc-816, Santa Cruz) at 1:50 dilution was first used, followed by HOXA10 (ab191470, Abcam) at 1:100 dilution on the slides. It is followed by 1:500 Alexa Fluor 568 Donkey anti-rabbit IgG (cat#A10042, Invitrogen) and 1:500 Alexa Fluor 488 Donkey anti-Goat IgG (cat#A11055, Invitrogen) secondary antibodies applied to the slides. Slides were counterstained and mounted with coverslips using Vectashield mounting medium containing 4′,6′-diaminido-2-phenylindole (DAPI; Vector Laboratories, Burlingame, CA, USA). Images were captured using a Zeiss LMS780 Confocal microscope (Carl Zeiss Instruments, Oberkochen, Germany) at 40X magnifications.

### Cell Culture and Lentivirus Infection

Three testicular cancer cell lines, TCam2, NT-2 and NCCIT, were generously provided by Dr. Leendert H. J. Looijenga (University Medical Center Rotterdam, Netherlands). Cell culturing methods followed the protocol described by Dr. Looijenga group (26). Lentiviurs vectors encoding control or *HOXA10* were constructed using the FUGWBW vector as a backbone (25, 27–29). Invitrogen gateway system was used to package lentivirus. Briefly, 3 μg of each lentiviral vector (*pFUGWBW* mock vector, *Flag-HOXA10*) together with 9 μg of the ViraPower packaging mix (Invitrogen) were transfected into 293T cells, using Lipofectamine 3,000 at 37°C and 5% CO2. Lentiviral particles were harvested by removing medium 48 h after transfection and used to infect TCam2, NT-2, and NCCIT cell lines. Lentivirus transduced cells were then exposed to 10 ng/ul blasticidin to select stably infected cells for 3 weeks. Polyclonal and antibiotic resistant cells were pooled for further experiments.

### Whole Transcriptome Profiling

Total RNA was extracted from TCam2 cells infected with *HOXA10* or control by using the mirVana RNA Isolation Kit (Ambion, Burlington, Canada) according to the manufacturer's protocol. Subsequent sequencing and analysis were described previously (30). In summary, the quantity and quality of the RNA samples were assessed by Nano-drop 2,000 as well as Agilent 2,100 Bio-analyzer (Caliper Technologies Corp., Canada) before sent for AmpliSeq Transcriptome Sequencing. Library preparation, sequencing, and primary analyses were performed by the UBC-DMCBH Next Generation Sequencing Center following the protocol described by Li et al. (31). Primary and differential gene expression analyses were carried out by the AmpliSeq RNA plugin available through the Ion Torrent™ suite Software (31) and the R/Bioconductor package DESeq2 (32).

### BrdU Incorporation and Cell Cycling Assays

Cell proliferation rates were measured by using the bromodeoxyuridine (BrdU) proliferation assay kit (Millipore, Catalog # 2750) according to the manufacturer's protocol and as previously described (29). Cell cycling was analyzed by FACS with 40 ug/mL propidium iodide staining following the protocol http://www.meduniwien.ac.at/user/johannes.schmid/ PIstaining3.htm. Relative DNA contents from 10,000 cells were analyzed by FACSCanto II flow cytometer and BD FACSDiva software v5.0.3 (Becton Dickinson) as we reported (25).

### Western Blotting

Cells were lysed in buffer [50 mM Tris-HCl, pH 7.4; 10 mM EDTA; 5 mM EGTA; 0.5% NP40; 1% Triton X-100 plus protease inhibitor (Roche)]. Protein concentration was measured by Pierce BCA protein assay kit (Thermo). Protein samples (40–60 ug) were mixed with SDS-PAGE loading dye, boiled and loaded on SDS-PAGE gel. After transferring onto PVDF membranes, proteins were blotted with primary antibody in TBST (50 mM Tris/pH 7.5, 0.15 M NaCl, and 0.05% Tween-20) plus 5% fat-free milk, washed and then with the HRP-conjugated IgG for 1 h. Membranes were then treated with ECL reagent (GE healthcare) and exposed to x-ray film. Vinculin, tubulin or histone H3 was used as a loading control. Antibody and primer information were included in Table S2.

## Results

### HOXA10 Expression in Testicular Tissues

**Figure 1:**
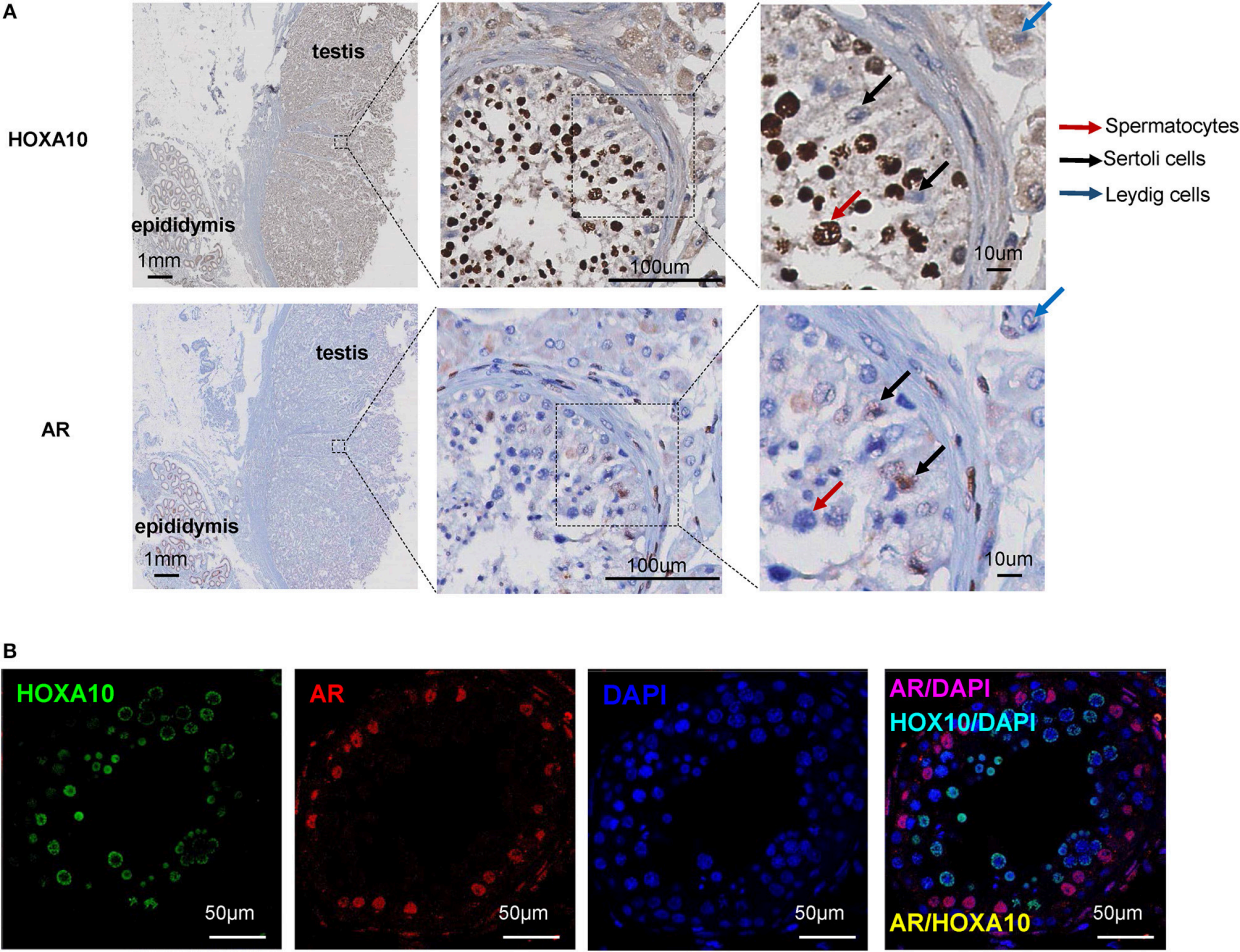
HOXA10 expression in benign testis **(A)** Whole sections of human testis and epididymis slides were stained with HOXA10 and AR antibodies. Spermatocytes, Sertoli cells, and Leydig cells were marked by arrows. **(B)** Co-immunofluorescence staining was used to localize HOXA10 (green) and AR (red) expression in cells within the seminiferous tubule.

To investigate the cellular localization of HOXA10 protein in testis tissue, the whole sections of benign testis slides were incubated with a HOXA10 antibody and HOXA10 expression was evaluated by immunohistochemistry (IHC). Within the seminiferous tubule, HOXA10 was highly expressed in the nuclei of spermatocytes at various differentiation stages (Figure 1A). In contrast, Sertoli cells were HOXA10 negative. Since Sertoli cells are often difficult to be distinguished from spermatocytes, we used the androgen receptor (AR) as a Sertoli cell-specific marker. Co-immunofluorescence assays with both HOXA10 and AR antibodies (Figure 1B) showed that HOXA10 (green) was not co-localized with the AR (red) in cells within the seminiferous tubules. Interestingly, HOXA10 was expressed in the cytoplasm of Leydig cells and absent in the smooth muscle cells surrounding the seminiferous tubules. On the same slides, HOXA10 was expressed at a relatively high level in the cytoplasm of the columnar cells and basal cells within the pseudostratified columnar epithelium of the epididymis, whereas the cells within the connective tissue underneath pseudostratified columnar were HOXA10 negative (Figure S1). These IHC results indicate that HOXA10 protein is expressed specifically in the nuclei of the spermatocytes (germline cells), while absent or localized in the cytoplasm of somatic cells including the Leydig cells in the testis and the pseudostratified columnar epithelial cells in the epididymis. Since HOXA10 is widely known as a transcriptional factor and our results show that HOXA10 is only localized in the nuclei of spermatocytes, this protein may be crucial in regulating the normal course of spermatocyte development and differentiation.

### Reduced Nuclear Expression of HOXA10 Protein in Testicular Cancers

**Figure 2:**
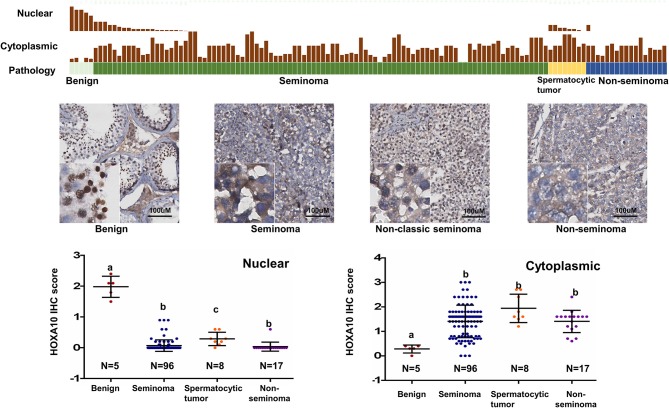
Loss of nuclear HOXA10 expression correlates with testicular tumors TGCT TMAs were described in the materials and methods section. They were used for immunohistochemistry analyses to evaluate HOXA10 protein expression. Both nuclear and cytoplasmic expression of HOXA10 protein levels were scored and plotted.

To study the expression of HOXA10 in testicular tumors, we applied IHC on a tissue microarray (TMA), containing 5 benign, 96 seminoma, 8 spermatocytic tumor and 17 non-seminoma (Figure 2). While highly expressed in spermatocytes of benign testes (HScore = 1.98 + 0.15), all cancerous testes had extremely low or no nuclear HOXA10 expression with HScores = 0.067 + 0.019 in seminoma, 0.207 + 0.06 in spermatocytic tumor, and 0 in non-seminoma. In contrast, cytoplasmic HOXA10 was mainly expressed in testicular cancer cells (HScore = 1.40 + 0.06 in seminoma, 1.67 + 0.167 in spermatocytic tumor and 1.45 + 0.13 in non-seminoma), but less commonly in benign testis (HScore = 0.28 + 0.07). We have also performed RNA *in situ* hybridization (RISH) assays using a *HOXA10*-specific RNA probe on the same TMA. We observed that both benign and cancerous testis tissues expressed *HOXA10* RNAs and there were no differences between benign and cancer tissue samples (Figure S2). Together, these results indicate that loss of nuclear expression of HOXA10 is associated with testicular cancers.

### Profiling HOXA10 Transcriptome in Testicular Cancer Cells

To study the cellular functions of HOXA10 in testicular cancer cells, we used three available TGCT cell lines, TCam2, NT-2, and NCCIT. While all cell models were *HOXA10* negative at both mRNA and protein levels (Figure S3), exogenous HOXA10 localized only in the nuclei. These results were consistent with our IHC observations that showed loss of nuclear expression of HOXA10 in all subtypes of testicular tumors. However, the differences of cytoplasm expression of HOXA10 between tumor cells and cell lines may reflect their different *in vivo* and *in vitro* growth conditions. Nevertheless, the expression pattern between tumor cells and *in vitro* cell models support that loss of nuclear function of HOXA10 is associated with testicular cancer.

**Figure 3:**
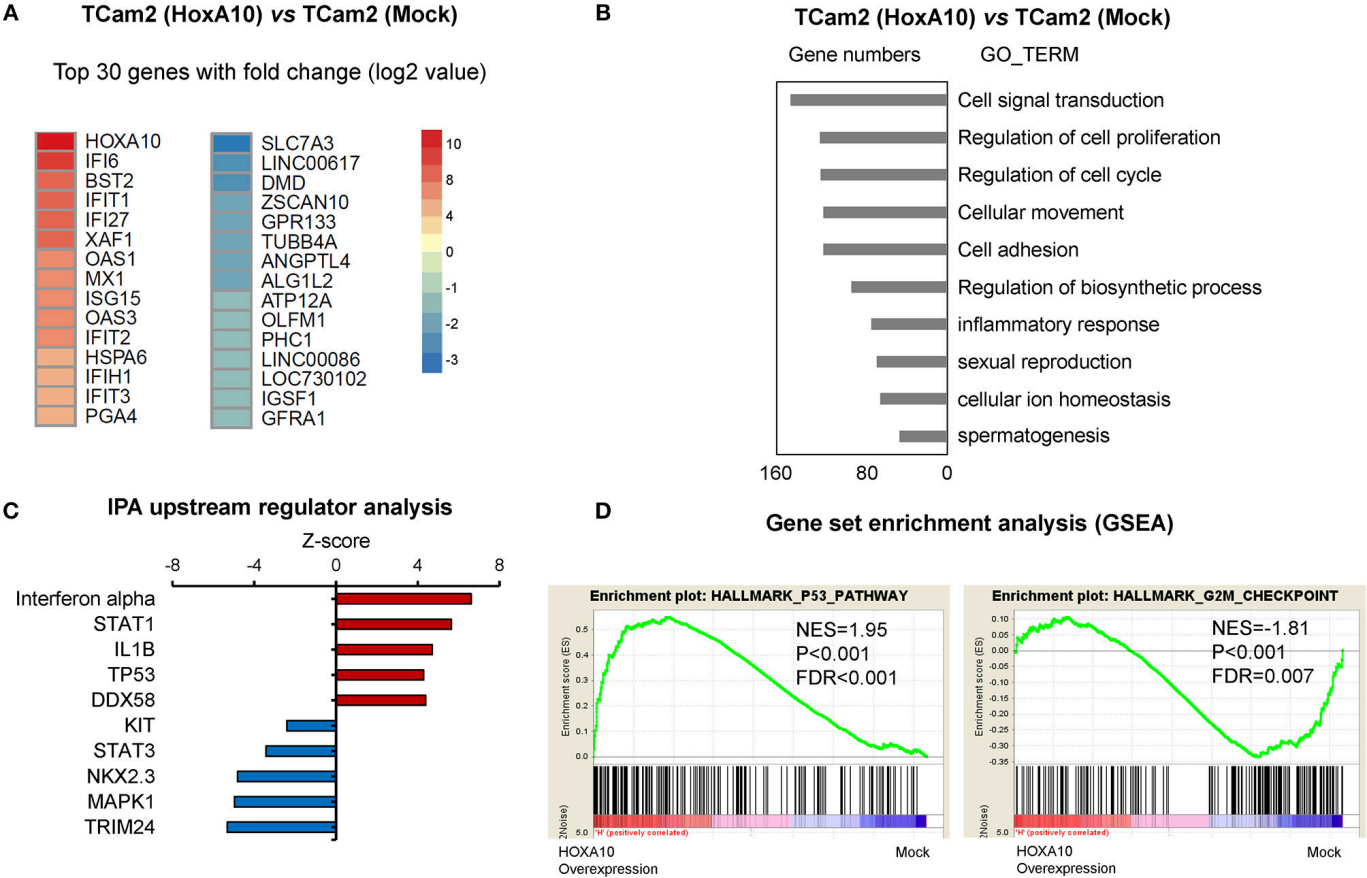
HOXA10 transcriptome in testicular cancer cells **(A)** A heatmap presents the top-ranked 30 genes that are transcriptionally regulated by HOXA10 in TCam2 cells. **(B)** Differentially expressed genes by HOXA10 were subjected to DAVID (version 6.7) analyses. Top ranked GO_TERM groups by *p*-value from the smallest to the largest are listed. **(C)** IPA predicts the upstream regulators of HOXA10 transcriptome. Red and blue nodes indicate signaling that is either stimulated or inhibited according to the Z-score. **(D)** HOXA10 transcriptomes were analyzed by the DESeq2 package in R. GSEA enrichment plots showed positive correlations of HOXA10-regulated genes with the TP53 pathway (normalized enrichment score (NES) = 1.95, false discovery rates (FDR) < 0.001) and negative correlation with the G2/M checkpoint (normalized enrichment score (NES) = −1.81, false discovery rates (FDR) = 0.007).

Since seminoma accounts for approximately half of testicular cancers, we used the TCam2 testicular seminoma cell line as a model to further characterize *HOXA10* transcriptome in testicular cancer cells. Using the AmpliSeq Transcriptome Sequencing method, we have identified 1,543 significantly upregulated genes and 839 downregulated genes by *HOXA10* (fold-change cutoff >1.5 and padj < 0.05). The 30 top ranked *HOXA10-*regulated genes by fold change are listed in Figure 3A. DAVID (Database for Annotation, Visualization, and Integrated Discovery) analyses indicated that overexpression of *HOXA10* was associated with gene ontology (GO) terms such as cell signal transduction, regulation of cell proliferation, cell cycling, cellular movement, and adhesion (Figure 3B). These results suggest that aberrant *HOXA10* expression may interrupt typical spermatocyte differentiations through altering cell cycles and cell proliferation, resulting in the development of TGCT. Similarly, Ingenuity Pathway Analysis (IPA) predicted that the upstream regulators of *HOXA10* included increased IFNα, STAT1, TP53 signaling and decreased STAT3 signaling, all of which have been implicated in tumor development (Figure 3C). We further applied Gene Set Enrichment Analysis (GSEA) to show that *HOXA10* transcriptome in TGCT cells was positively associated with TP53 pathway (NES = 1.95, FDR *q* < 0.001) while negatively associated with G2/M checkpoint (NES = −1.81, FDR *q* < 0.007) (Figure 3D). These results support that HOXA10 suppresses TGCT cell proliferation through the modulation of the TP53 signaling. Based on these results, we hypothesize that HOXA10 can enhance the functions of TP53 to suppress TGCT cell proliferation.

### HOXA10 Suppresses Testicular Cancer Cell Proliferation

**Figure 4:**
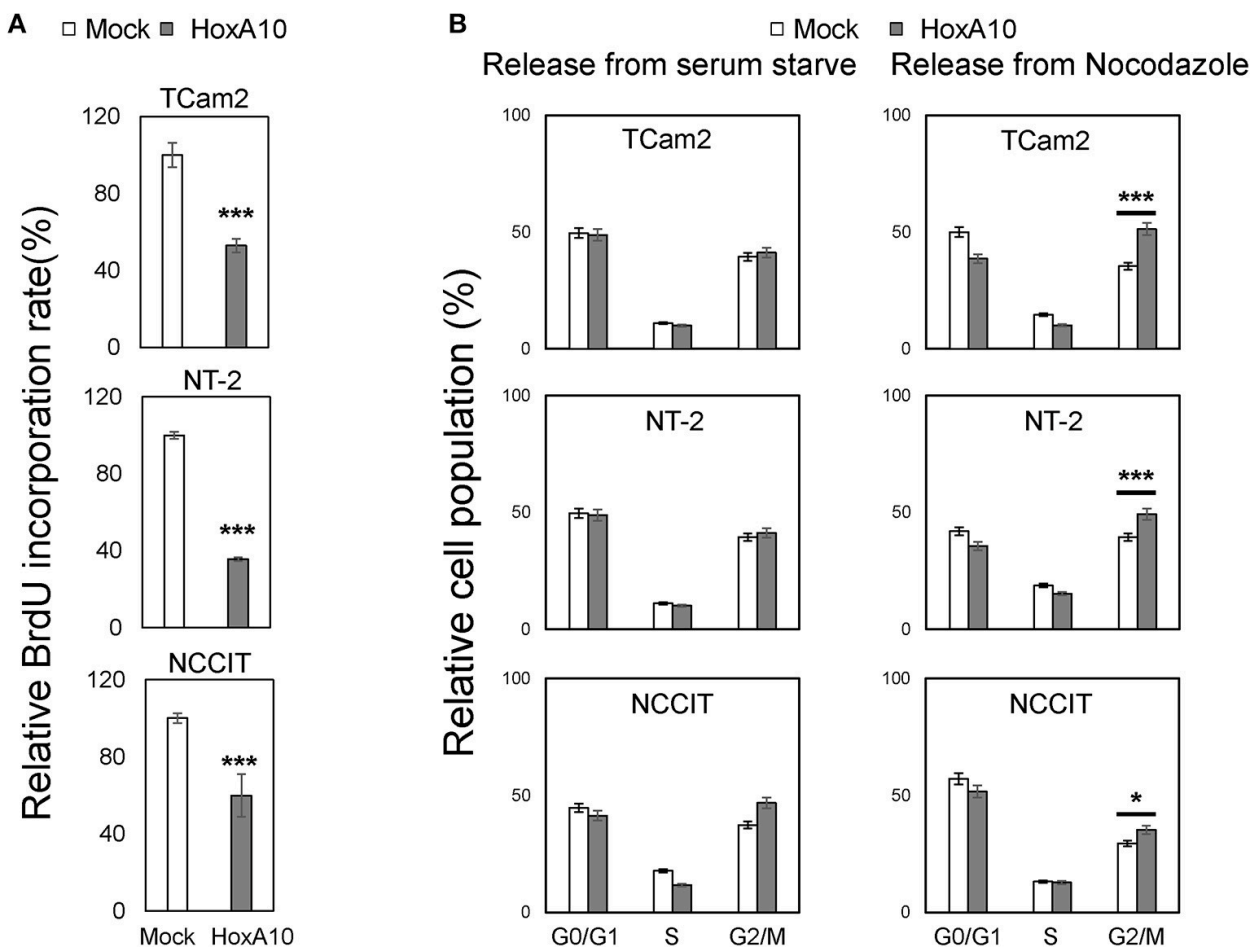
HOXA10 inhibits testicular cell proliferation **(A)** TCam2, NT-2, and NCCIT cell lines with control or HOXA10 overexpression were used to perform BrdU incorporation assays to measure cell proliferation rates. BrdU results represent colorimetric quantitative measurements (OD at 450 nm) of cellular BrdU incorporation into DNA. *p < 0.05 and ***p < 0.001. **(B)** TCam2, NT-2, and NCCIT cells with control or HOXA10 overexpression were synchronized by serum starvation for 12–16 h and then either released from serum starved conditions (left); TCam2, NT-2, and NCCIT cells were synchronized by Nocodazole, then released for cell cycling progression (right). Cells were collected and subjected to FACS assays to measure cell populations at the G0/G1, S, and G2/M phases.

To confirm the transcriptomic analyses results, we performed BrdU incorporation assays to show that HOXA10 overexpression suppressed cell proliferation of all three TGCT cell models. These results suggested that our findings from the transcriptomic studies in TCam2 cell may also be applied to the non-seminoma NT-2 and NCCIT cell models (Figure 4A). To delineate the specific cell cycling stages regulated by HOXA10, we performed FACS assays on TCam2, NT-2, and NCCIT cells (Figure 4B). Cells expressing control or HOXA10 were first serum starved to synchronize cell cycling at the G0/G1 stage, followed by adding culture medium containing 10% serum to release cell cycling. We observed that HOXA10 has no impact on cells passing from the G1 to S and G2/M phases. However, when cell cycling was synchronized at the G2/M phases by nocodazole and then released, HOXA10 caused significant delays of cell cycling passing through the G2/M phases into the G1 phase. These results indicate that HOXA10 inhibited TGCT cell proliferation through impeding the G2/M progression.

### HOXA10 Mediates Multiple Signaling to Regulate Testicular Cell Proliferation

**Figure 5:**
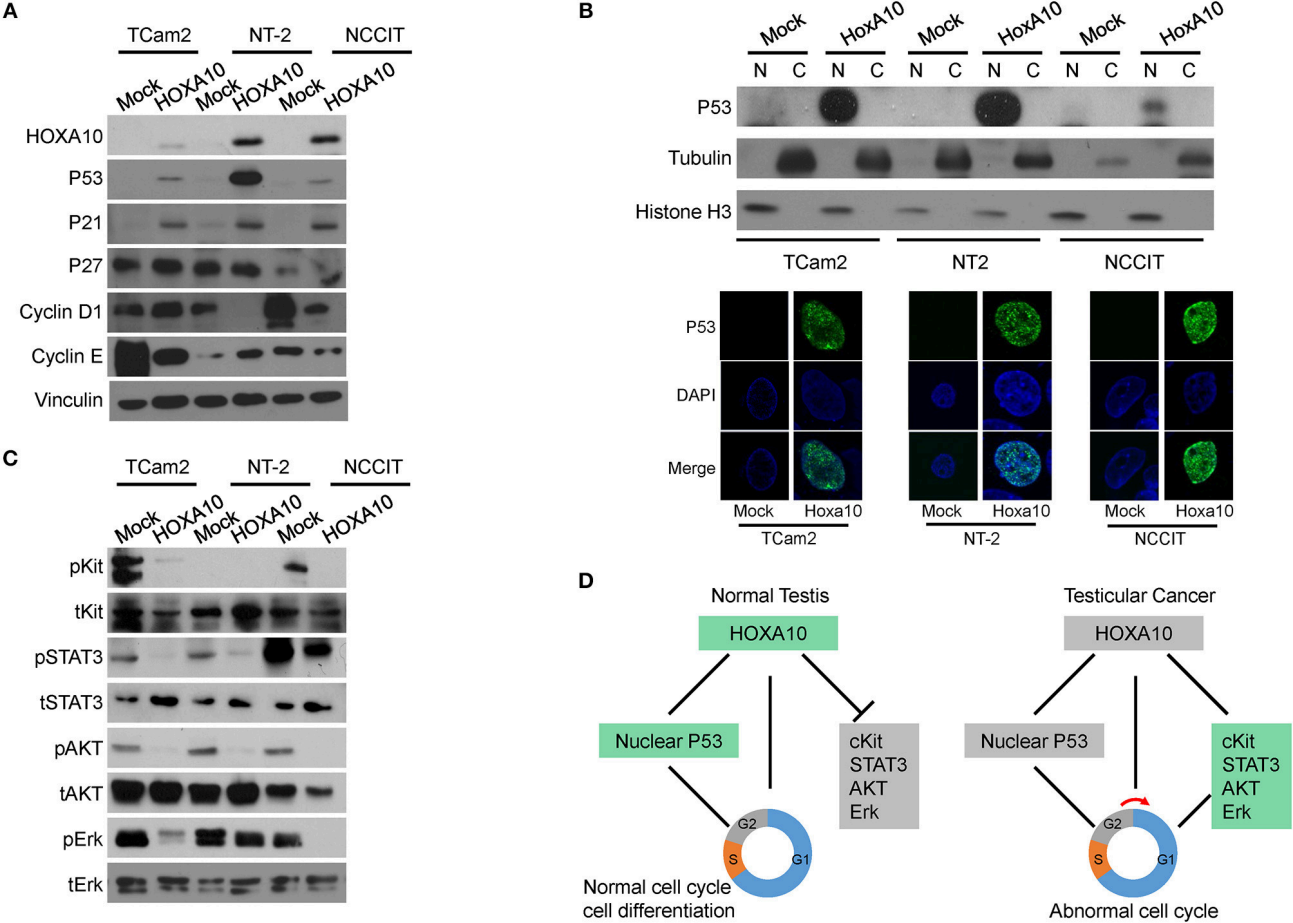
HOXA10 mediates multiple signaling to regulate testicular cell proliferation **(A)** Protein lysates from TCam2, NT-2, and NCCIT cells with control or HOXA10 overexpression were used to perform immunoblotting with HOXA10, TP53, P21, P27, Cyclin D, and E antibodies, using Vinculin as the loading control. **(B)** Nuclear (N) and cytoplasmic (C) fractions of TCam2, NT-2, and NCCIT cells were extracted and used to detect TP53 expression by immunoblotting with tubulin and Histone H3 as loading control. TP53 subcellular localization in testicular cells were also detected by immunofluorescence microscopy. **(C)** Protein lysates from TCam2, NT-2, and NCCIT cells with control or HOXA10 overexpression were used to perform immunoblotting with total and phosphorylated Kit, STAT3, AKT, and Erk. Note: all of the experiments were repeated three times and only one representative repeat was presented. **(D)** A schematic illustration of the proposed model of HOXA10 and its relationship with TP53 in promoting the development of testicular cancer.

To further confirm the predicted HOXA10 functions by transcriptomic analyses and to better understand the signaling pathways regulated by HOXA10, we used immunoblotting assays in the three TGCT cell models. First, we observed that HOXA10 transfection induced TP53 expression (Figure 5A). Since TP53 mediates its tumor-suppressive activities through regulating gene transcriptions, we found that HOXA10-induced TP53 was localized in the nuclei via immunoblotting nuclear extracts of cells and immunofluorescence microscopy assays (Figure 5B). Furthermore, upregulation of TP53 by HOXA10 was concurrent with the induction of cyclin-dependent kinase inhibitor p21, but not p27 (Figure 5A). These findings are consistent with the relationship between p21 and TP53, whereby TP53-mediated growth inhibition is dependent on the induction of p21 to suppress cell cycle progression (33). Interestingly, cyclin D1 protein levels were reduced by HOXA10 in NT-2 and NCCIT but not the TCam2 cells, while cyclin E expression was suppressed by HOXA10 in TCam2 and NCCIT, but not the NT-2 cells. Nevertheless, HOXA10 stimulates the TP53 and putatively the TP53-p21 axis, thereby exerting general inhibitory effects to all TGCT cell models regardless the inconsistent changes in cyclin D and cyclin E expressions.

In addition to confirming the TP53 signaling, we also performed immunoblotting assays to validate the relationship between HOXA10 and cKit as well as STAT3 signaling pathways (Figure 5C). The protein cKit is a receptor tyrosine kinase generally localized on the cell surface. It can be activated by the stem cell factor, SCF, to trigger the downstream signaling to stimulate cell proliferation, survival, and migration (34). cKit was expressed in both TCam2 and NCCIT cells and its expression was suppressed by HOXA10. Moreover, the functionally active form of STAT3 (phosphorylated STAT3, pSTAT3) was observed in the three testicular cell lines, with relatively higher levels in the NCCIT cells. HOXA10 reduced pSTAT3 levels in the NCCIT cells, and abolished pSTAT3 expression in both TCam2 and NT-2 cells. Furthermore, phosphorylated AKT and Erk kinases as the downstream effectors of the cKit and MAPK1 pathways were also inhibited by HOXA10. These results indicate that HOXA10 not only stimulates the TP53-p21 axis, but also suppresses the cKit and STAT3 pathways to inhibit testicular cell proliferation (Figure 5D), supporting that loss of HOXA10 functions is associated with testicular cancers.

## Discussion

In this study, we report that HOXA10 is expressed in the nucleus of benign spermatocytes (Figure 1) and frequently loses its nuclear localization in TGCTs including both seminoma and non-seminoma (Figure 2). Traditionally, HOXA10 is considered a nuclear protein that functions as a transcription factor to regulate downstream gene expressions. To date, only one group reported cytoplasmic localization of HOXA10 via interacting with the histone deacetylase Sirtuin2 (SIRT2) (35). This report suggested that SIRT2 can bind with HOXA10 in the cytoplasm to suppress HOXA10-mediated transcription. Similarly, we also observed extensive cytoplasmic HOXA10 expressions from IHC staining in TGCT patient samples (Figure 2), despite similar levels of RNA expressions between benign and cancerous tissues as further cross-validated with the RISH procedure (Figure S2). Specific mechanisms leading to this change in HOXA10 localization remain elusive. One possible explanation may be due to increased SIRT2 that sequesters HOXA10 in the cytoplasm. In addition, since HOXA10 tyrosine phosphorylation was reported to have reduced its DNA-binding activities (36), disruptions in HOXA10 phosphorylation regulators such as JAK2 (37) and phosphatases SHP1/SHP2 may contribute to the cytoplasmic localization (38). Other putative explanations include reduced microRNAs that regulate HOXA10 expression and genomic mutations that affect HOXA10 nuclear localization sequences. These two theories are unlikely to be true as the former should lead to significant change in HOXA10 protein abundance inconsistent with our observations in this study and the latter has not been reported from recent genome-wide association studies (GWAS) (39).

Changes in HOXA10 localization in TGCT has functional significances. Our transcriptomic studies showed that overexpression of “functional” HOXA10 in the nucleus of TCam2 cells (Figure S3) led to changes in the differential expression of numerous genes (Figures 3A–C), notably the stimulation of TP53 pathway and the suppression of G2/M checkpoint signaling (Figures 3C,D). These associations were confirmed with our molecular analyses (Figures 4, 5A,B), suggesting an anti-proliferative property of HOXA10 in our cell models. For the TP53 signaling, previous reports have shown that not only *TP53* promoter contained HOXA binding sites, but also that HOXA10 was a positive regulator of *TP53* in breast cancer cells (21). Interestingly, this anti-tumorigenic HOXA10-TP53 relationship has only been described in breast cancer but not other types of cancer, raising the question of whether this pathway is only specific to endocrine or germ-related tumors. With regard to the G2/M checkpoint, the suppressive effect of HOXA10 on G2/M checkpoints may or may not be related to TP53. Although HOXA10-induced TP53 may directly inhibit G2/M via cdc2 (40), it is possible that the suppressive effects of HOXA10 on other signaling pathways such as p21, cKit, STAT3, and MAPK also play a role in this process. The latter mechanisms are more likely to be true at least in NCCIT cells, which have mutated TP53 in contrast to that of TCam2 and NT-2 cells (41). In fact, as a transcription factor acting on numerous downstream effectors, whether HOXA10 directly or indirectly regulates signaling pathways involving proteins such as p21, cKit, STAT3, AKT, and Erk remains elusive. It is also possible that, in the setting of TGCT, the aberrant cytoplasmic localization could lead to new functional effects of HOXA10 in the cytoplasm. These hypotheses warrant further investigations. Loss of nuclear HOXA10 may also provide insights into differentiation in TGCTs. Somatic cancers usually develop via entering an undifferentiated state for clonal evolution and expansion. As a gene known to play important roles in development, HOXA10 is expected to affect gene expressions related to cell differentiation. However, TGCT is a special case as it is relatively undifferentiated and develops from the malignant counterpart of undifferentiated germ cells (e.g., gonocytes or primordial germ cells). A representative example is seminoma, which predominantly presents in a state of “blocked” differentiation (8). As a result, (de)differentiation may play less critical roles than proliferation in the development of TGCT. In this context, our findings suggest that HOXA10 may have more impact on suppressing proliferation rather than regulating differentiation in anti-TGCT tumorigenesis. Nevertheless, since HOXA10 has numerous downstream effectors, it is also possible that HOXA10 is required for normal spermatocyte differentiation to prevent tumorigenesis. One limitation of this study might be related to the TGCT tumor models. We have used all three TGCT cell lines that are currently available. The TCam2 cell line is the only one that resembles seminomas both in genomic and epigenetic changes (26). However, TCam2 cells have mutated BRAF genes uncommonly seen in TGCT tumors. While it was hypothesized that the constitutively active BRAF gene enables TCam2 cells to be propagated *in vitro*, this aberration may interfere with cell growth and proliferation, particularly related to the RAS signaling pathway (e.g., Erk) (42). On the other hand, NCCIT cells resemble teratocarcinoma whereas NT-2 cells are derived from malignant embryonal carcinoma (43, 44). Comprehensive genetic and epigenetic characterizations of these cell lines may provide further insights into whether these models resemble the average non-seminomas.

Although our study is the first to report clinical and functional significances of *HOXA10* in testicular cancer, it is interesting that *HOXA10* mutations were reported to be linked to higher incidence of inguino-scrotal cryptorchidism (45, 46) and that cryptorchidism is one of the strongest risk factors for testicular cancer (7). In fact, although weaker, cryptorchidism remains a risk factor for TGCT even after early orchiopexy interventions (47). These finding, together, suggest that TGCT tumorigenesis likely happens both *in utero* and after birth, and that dysfunctional HOXA10 may aid the tumorigenic process throughout development. In addition, our findings may also have implications for potential biomarkers. The current TGCT biomarkers of AFP, b-HCG, and LDH have a relatively low sensitivity and specificity (48). New tumor markers, particularly miRNAs have been actively developed (49, 50). The expression levels of HOXA10 are unlikely to be helpful since the absolute expression of HOXA10 largely remains unchanged in TGCT. However, as HOXA10 frequently loses its canonical functions in TGCT, it might be worthwhile to investigate whether downstream effectors of HOXA10 are amenable to non-invasive detections clinically.

In summary, we report that altered HOXA10 localization is associated with TGCT and that loss of nuclear HOXA10 canonical function results in attenuated TP53 signaling and increased cell cycling through regulating the G2/M checkpoint. Functional studies also further confirm that HOXA10 has anti-proliferative activities in TGCT cells putatively via signaling pathways entailing TP53, cKit, STAT3, MAPK.

## Author Contributions

RC, HL, and YL contributed to the conception and design of the study as well as data acquisition and interpretation. RC drafted the manuscript. LF evaluated and scored IHC staining. MG, LN, and XD reviewed the manuscript critically. XD supervised and designed the study. All authors contributed to interpretation of findings, reviewed, edited, and approved the final manuscript.

### Conflict of Interest Statement

The authors declare that the research was conducted in the absence of any commercial or financial relationships that could be construed as a potential conflict of interest.
