# Supplementary material for: Loss of Nuclear Functions of HOXA10 Is Associated With Testicular Cancer Proliferation
Source: Front Oncol. 2018 Dec 7;8:594. doi: 10.3389/fonc.2018.00594 (PMC6292994; doi:10.3389/fonc.2018.00594)
Supplement: Supplementary file 1 [file Data_Sheet_1.docx]

***Supplementary Material***

**Loss of Nuclear Functions of HOXA10 is Associated with Testicular Cancer Proliferation**

**Ruiqi Chen^1,2^, Haolong Li^1^, Yinan Li^1^, Ladan Fazli^1^, Martin Gleave^1^, Lucia Nappi^1^, Xuesen Dong^1*^**

*** Correspondence:**

Xuesen Dong, PhD
E-mail: [xdong@prostatecentre.com](mailto:xdong@prostatecentre.com)

**
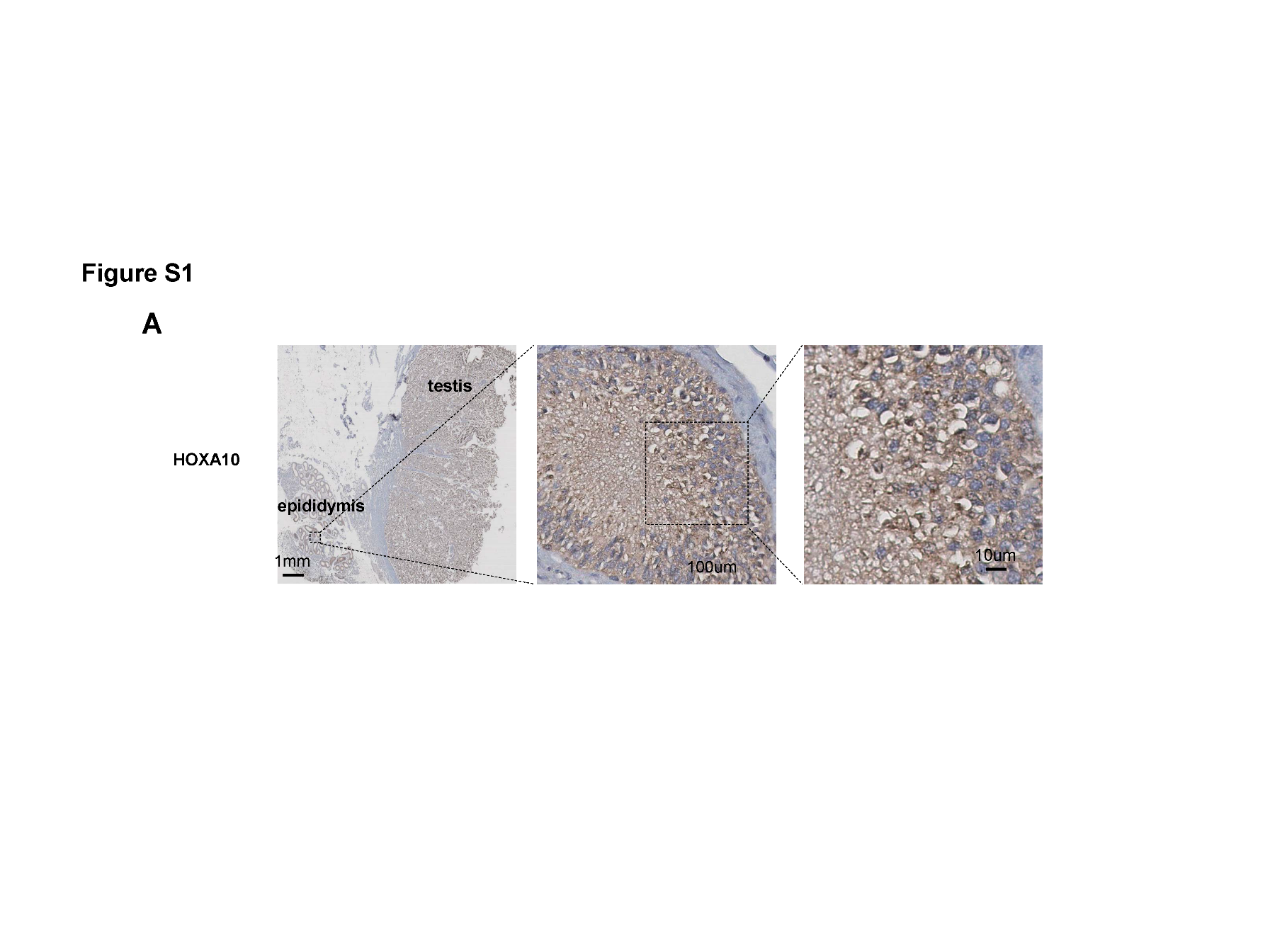
**

**Figure S1** Whole sections of human testis and [epididymis](http://www.siumed.edu/~dking2/erg/epidid.htm) slides were stained with the HOXA10 antibody.

**
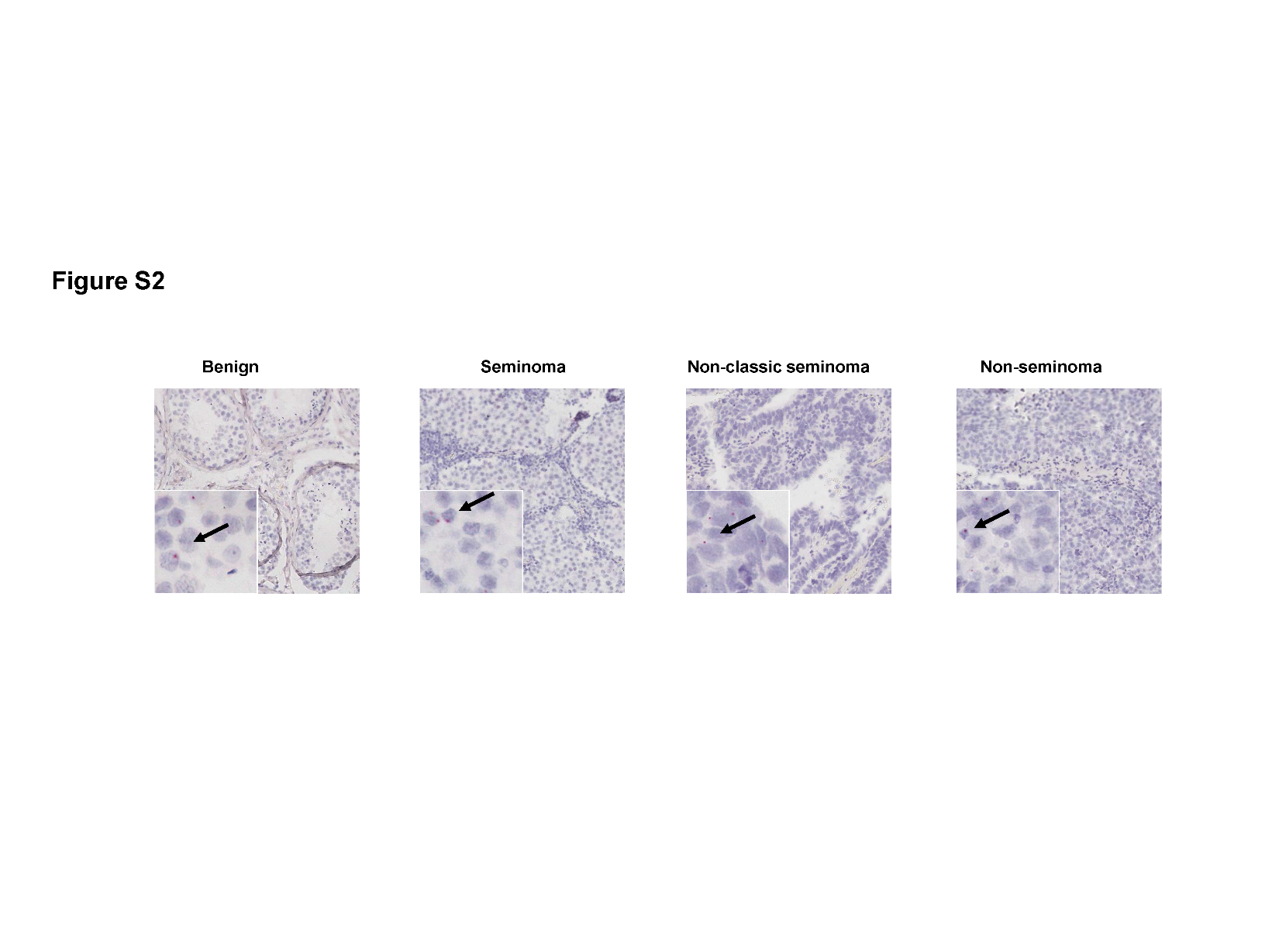
**

**Figure S2** RNA in situ hybridization (RISH) using a HOXA10 specific probe was performed on the TGCT TMA. Representative images were shown. HOXA10 RISH signaling was presented as red dots marked by arrows.

**
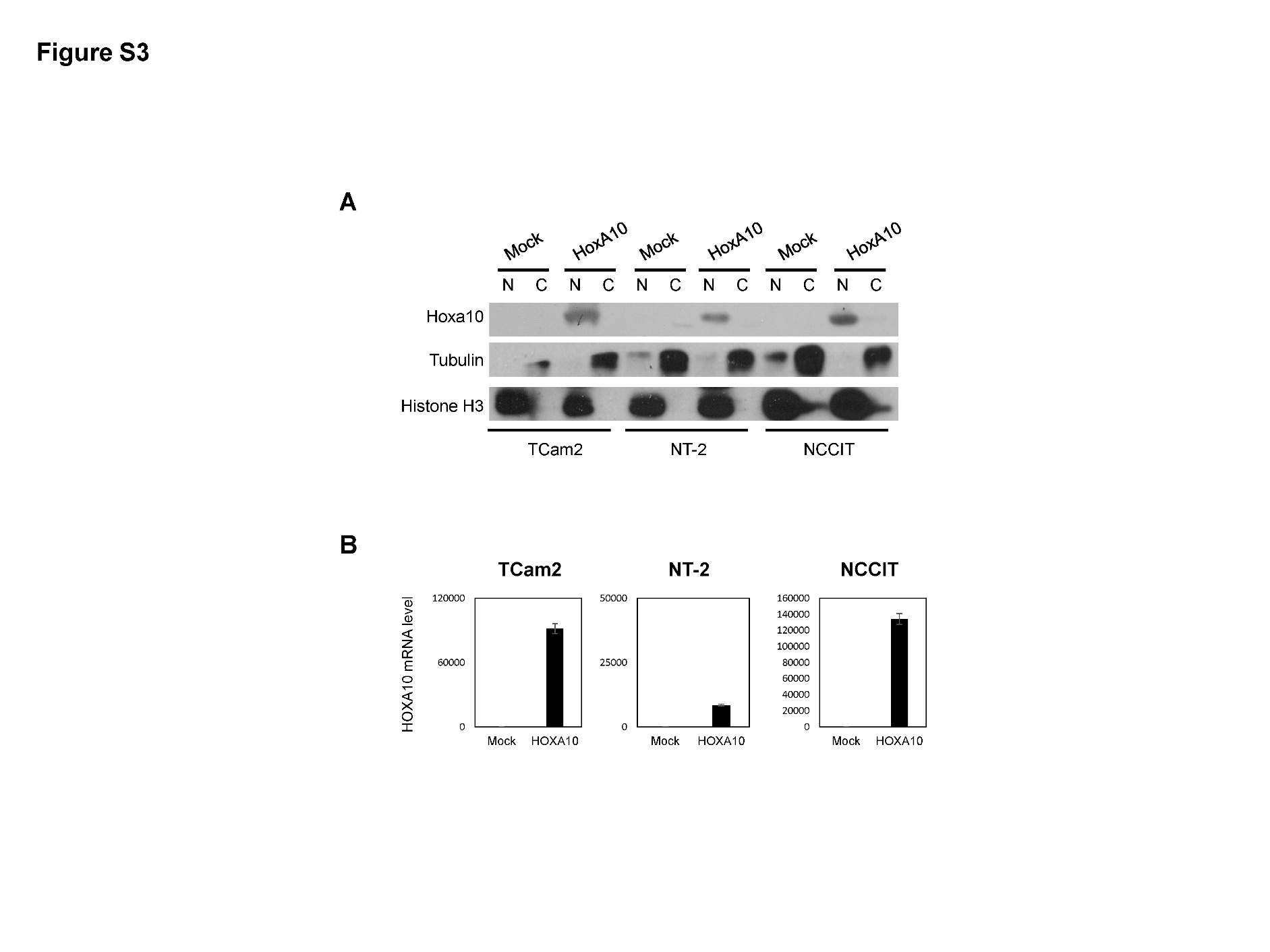
**

**Figure S3** TCam2, NT-2, and NCCIT cell were infected by lentivirus encoding control or HOXA10. After blasticidin selection, HOXA10 protein and mRNA expressions were validated by immunoblotting and real-time PCR assays.
